# Phenotypic and genotypic characteristics of carbapenem-resistant *Enterobacteriaceae* in a tertiary-level reference hospital in Turkey

**DOI:** 10.1186/s12941-016-0136-2

**Published:** 2016-04-06

**Authors:** Irmak Baran, Neriman Aksu

**Affiliations:** Department of Microbiology, Ankara Numune Training and Research Hospital, Talatpaşa Bulvarı No:5 Altındağ, 06100 Ankara, Turkey; Esat Caddesi 101/3 Küçükesat, 06660 Ankara, Turkey

**Keywords:** *Enterobacteriaceae*, Carbapenem resistance, OXA-48, NDM-1, VIM, Modified Hodge test, Multiplex PCR, Multidrug resistance

## Abstract

**Background:**

*Enterobacteriaceae* are among the most common pathogens that are responsible for serious community-acquired, hospital-acquired, and health-care associated infections. The emergence and spread of carbapenem-resistant *Enterobacteriaceae* (CRE) have become an increasing concern for healthcare services worldwide. Infections caused by these bacteria have been associated with significant morbidity and mortality and treatment options have been limited. The rapid and accurate detection of carbapenem resistance in these bacteria is important for infection control. The aim of this study was to investigate the phenotypic and genotypic features of CRE strains isolated in a tertiary-level reference hospital in Turkey.

**Methods:**

A total of 181 CRE strains were included in the study. Antimicrobial susceptibility rates were tested using Vitek 2 system. Modified Hodge test (MHT) was performed using meropenem and ertapenem discs. Metallo-β-lactamase antimicrobial gradient test (E-test MBL strips) were used for evaluation of metallo-β-lactamase production. A multiplex PCR was used for detection of carbapenems resistance genes (IMP, VIM, KPC, NDM-1 and OXA-48).

**Results:**

The OXA-48 gene was detected in 86 strains, NDM-1 gene in six strains, VIM gene in one strain. IMP and KPC genes were not identified. Three strains produced both OXA-48 and NDM-1 and one strain produced both OXA-48 and VIM. In two patients more than one genus of OXA-48 positive CREs was isolated. Ninety-two of the isolates were multidrug-resistant. One hundred and ten isolates were MHT with meropenem (MEM-MHT) positive and 109 isolates were MHT with ertapenem (ERT-MHT) positive. Nine of the isolates were positive with E-test MBL strips. The sensitivity of MEM-MHT and ERT-MHT for detection of OXA-48 was 70.9 and 70.6 %, respectively. MEM-MHT was found highly discriminative for OXA-48 *Escherichia coli* (p < 0.001). The sensitivity of E-test MBL for NDM-1 was 66.7 %. A statistically significant correlation was observed between OXA-48 gene and MHT positivity and between NDM-1, VIM gene and E-test MBL positivity (p < 0.001).

**Conclusions:**

OXA-48 gene is spreading rapidly to many different species of *Enterobacteriaceae* in the hospital environment. While OXA-48 is still the most common source of carbapenem resistance in *Enterobacteriaceae* in our country, NDM-1 is increasingly being isolated from patients without a history of foreign contact.

## Background

*Enterobacteriaceae* are common human pathogens and colonizers of the human intestinal tract which can cause a broad range of diseases including urinary tract infections, pneumonia, bloodstream infections, intra-abdominal, skin and soft tissue infections in both community and hospital settings [1–4]. Antibiotic resistance in *Enterobacteriaceae* is an important public health problem. Because these microorganisms are associated with increased length of hospital stay, the cost of treatment, mortality and morbidity [5–8].

Dissemination of infections caused by extended-spectrum β-lactamases (ESBL) and AmpC β-lactamases-producing *Enterobacteriaceae* has compromised susceptibility to cephalosporins in many areas of the world and has led to increased use of carbapenems which are stable antibiotics against these enzymes. However widespread use of carbapenems resulted in the emergence of carbapenem-resistant *Enterobacteriaceae* (CRE) [3, 4, 6, 9, 10].

Mainly two different mechanisms are responsible for carbapenem resistance in *Enterobacteriaceae*: (i) hyper production of ESBL or AmpC enzymes combined with porin loss or alteration or upregulated efflux pump and/or (ii) production of carbapenem-hydrolyzing carbapenemases [1, 6, 11–13].

In 1993, NmcA in *Enterobacter cloacae* was identified as the first carbapenemase enzyme in *Enterobacteriaceae* [4, 10]. Since then, outbreaks caused by carbapenemase-producing *Enterobacteriaceae* (CPE) have been reported from various regions of the world [1, 4, 14].

Carbapenemases are categorized in Ambler classification system as follows, the class A carbapenemases (GES, KPCs) which are inhibited by clavulanic acid; the class B or metallo-β-lactamases (VIM, IMP, NDMs) which are inhibited by ethylene diamine tetra-acetic acid (EDTA); and the class D oxacillinases which are not affected by clavulanic acid or EDTA [3, 7, 8, 14, 15].

Plasmid-mediated carbapenemase production is the most important and common cause of carbapenem resistance in CRE [3, 7, 9, 12, 16]. The emergence of plasmids containing carbapenemase genes was considered as a serious health threat because it may ease the spread of carbapenem resistance [17]. Also, carbapenem-resistant isolates frequently carry additional antimicrobial resistance genes to other classes of antibiotics including fluoroquinolones, aminoglycosides, trimethoprim—sulfamethoxazole, and this limits the treatment options [1, 6, 9, 10, 13, 18].

A limited number of published data from our country about carbapenem resistance in *Enterobacteriaceae* other than carbapenem-resistant *Klebsiella pneumonia* (CRKP) are available.

The aim of this study was to investigate phenotypic and genotypic characteristics of carbapenem resistance in CRE isolated from patients in our hospital.

## Methods

### Setting and study design

This prospective study was conducted over a period of 13 months between January 31st 2013–February 14th 2014 in Medical Microbiology Laboratory of Ankara Numune Research and Training Hospital which is a 1200 bed capacity tertiary-level reference hospital with surgical, medical, orthopaedic and intensive care units and serves not only a population of 4,000,000 inhabitants but also the surrounding cities.

In this period, a total of 6426 *Enterobacteriaceae* species were isolated from different clinical specimens (blood, urine, wound, tracheal aspirate, abscess, etc.) sent to our laboratory. Clinical samples were analysed with standard laboratory techniques.

### Bacterial strains and antimicrobial susceptibility testing

All *Enterobacteriaceae* isolates at the time of isolation were identified using Vitek 2 automated system (bioMérieux, France) with ID-GNB card and then confirmed by MALDI Biotyper (Bruker, Germany). Antibiotic susceptibility tests were done using vitek AST-GN cards. Susceptibilities to ampicillin (AMP), amoxicillin-clavulanic acid (AMC), piperacillin-tazobactam (TZP), cefuroxime (CXM), cefuroxime-axetil (CAE), cefoxitin (FOX), ceftazidime (CAZ), ceftriaxone (CRO), cefepime (FEP), ertapenem (ERT), imipenem (IPM), meropenem (MEM), amikacin (AMK), gentamicin (GEN), ciprofloxacin (CIP), colistin (CST), trimethoprim-sulfamethoxazole (SXT) were tested. CLSI 2013 M100-S23 breakpoint values were used [19]. For colistin the CLSI recommendation for *Acinetobacter* spp. were used [19, 20]. *Escherichia coli* ATCC 25922 and *Pseudomonas aeruginosa* ATCC 27853 were used as quality control strains.

Isolates were considered as CRE if they were found resistant or intermediate to one or more of the carbapenems (ERT, IPM and MEM). A total of 181 (2.82 %) isolates were identified as CRE and were included in this study. Minimal inhibitory concentration (MIC) of ERT, IPM and MEM were also tested by E-test. E-test results were interpreted according to the CLSI 2013 criteria [19].

### Phenotypic methods for detecting carbapenemase activity testing and ESBL production

Modified Hodge test (MHT) was used for screening carbapenemase production using ERT and MEM according to CLSI guidelines [19]. Metallo-β-lactamase production was investigated with metallo-β-lactamase antimicrobial gradient test which is IPM and IPM/EDTA E-test MBL strips (bioMérieux, France). A ratio of MIC_IPM_/MIC_IPM/EDTA_ ≥8 considered presumptive of metallo-β-lactamase. ESBL production was evaluated by CLSI screening and confirmatory test methods [19].

### Molecular analysis of carbapenemase genes

For investigation of carbapenemases genes a multiplex PCR (hyplex^®^ SuperBug ID, Amplex, Germany) was used. This system can identify metallo-β-lactamase genes such as bla_VIM(1–13)_, bla_IMP(1–22)_ and bla_NDM-1_, oxacillinase genes such as bla_OXA-48_-like encoding genes (including. bla_OXA-162_, _OXA-181, OXA.204_ and bla_OXA-244_) and all variants of bla_KPC(1-10)_ genes.

DNA was extracted from bacteria grew in 24-h culture. After PCR reactions, amplification products were heat-denaturated to obtain single-stranded DNA and loaded to microtiter plates coated with specific probes. Hybridization reaction principle was used to investigate carbapenemase gene loci in loaded DNA. Hybridization of complementary sequences was then detected using ELISA principle. The colour formed by the chromogenic reaction was then measured by a photometer at a wavelength of 450 nm.

### Statistical analysis

All data were evaluated by using SPSS for Windows version 11.5. We used Chi square test and Fischer’s exact test to relate genes and MHTs for categorical data, and we also calculated screening test results. Descriptive statistics, frequencies and percentages were given. The statistical significance level was set to 0.05.

## Results

### CRE isolates

During the study period 181 CRE were identified from 6426 *Enterobacteriaceae* isolates. The most common species were *Klebsiella pneumoniae* (69, 38.12 %), followed by *Serratia marcescens* (33, 18.23 %), *Morganella morganii* (17, 9.39 %), *Proteus mirabilis* (17, 9.39 %), *E. coli* (13, 7.18 %), *Enterobacter cloacae* (13, 7.18 %), *Enterobacter aerogenes* (10, 5.52 %), *Citrobacter freundii* (2, 1.1 %), *Raoultella planticola* (2, 1.1 %), *Proteus vulgaris* (1, 0.55 %), *Providencia rettgeri* (1, 0.55 %), *Providencia stuartii* (1, 0.55 %), *Cronobacter sakazakii* (1, 0.55 %), *Klebsiella oxytoca* (1, 0.55 %).

The majority of strains were isolated from internal medicine intensive care unit (ICU) (49, 27.07 %). This was followed by post anesthesia care unit (PACU) (18, 9.94 %), surgical ICU (10, 5.52 %), general surgery unit (10, 5.52 %), burn unit (9, 4.97 %), urology (9, 4.97 %), and emergency internal medicine (9, 4.97 %). Remaining isolates were isolated from other clinics. Most of the 181 isolates were collected from blood (59 isolates, 32.6 %), from urine (49, 27.07 %), and from wounds (31, 17.13 %). The remaining isolates were from tracheal aspirates (21, 11.6 %), abscess culture (9, 4.97 %), catheter (4, 2.21 %), pleural fluid (3, 1.66 %), peritoneal fluid (1, 0.55 %), bile (1, 0.55 %), sputum (1, 0.55 %), rectal swab (1, 0.55 %), cerebrospinal fluid (CSF) (1, 0.55 %). Table 1 shows distribution of *Enterobacteriaceae* species according to clinical samples.

**Table 1:** Species and specimen distribution of 181 carbapenem-resistant *Enterobacteriaceae*

Species	Specimen type (n)								
	Blood	Urine	Wound	Trachael aspirate	Abscess	Catheter	Pleural fluid	Other*	Total
*K. pneumoniae*	18	25	8	10	3	4		1	69
*S. marcescens*	29	1	1	1	1				33
*M. morganii*	2	3	9	2				1	17
*P. mirabilis*	3	7	5	2					17
*E. coli*	3	5	1		3			1	13
*E. cloacae*	1	4	4				3	1	13
*E. aerogenes*	1	2		4	2			1	10
*C. freundii*	1	1							2
*R. planticola*		1		1					2
*P. vulgaris*			1						1
*P. rettgeri*			1						1
*P. stuartii*			1						1
*C. sakazakii*	1								1
*K. oxytoca*				1					1
Total	59	49	31	21	9	4	3	5	181

*Bile, sputum, rectal swab, peritoneal fluid, cerebrospinal fluid

### Phenotypic tests for carbapenemase production and carbapenemase genes identified by multiplex PCR

When 181 CRE isolates were analyzed with MHT with MEM (MEM-MHT) and MHT with ERT (ERT-MHT) for carbapenemase production; 110 (60.8 %) isolates were found positive with MEM-MHT and 109 (60.2 %) isolates were positive with ERT-MHT. Only nine (5 %) of the isolates were tested positive with E-test MBL strips for metallo-β-lactamase production.

In 86 (47.51 %) of the isolates, the bla_OXA-48_ gene was detected by multiplex PCR. Six isolates (3.33 %) were bla_NDM-1_ gene positive. In one strain (0.56 %) bla_VIM_ gene was found positive. A breakdown of carbapenemase positive isolates is presented in Table 2. At least one carbapenemase-encoding gene was found positive in 90 (49.72 %) of the isolates. In four of the strains, multiple carbapenemase genes were identified. Three of the bla_NDM-1_ positive *K. pneumoniae* also possessed bla_OXA-48_ gene. In one of the *K. pneumoniae*, bla_VIM_ gene was detected alongside bla_OXA-48_ gene. The bla_IMP_ and bla_KPC_ genes were not detected in any of the isolates.

**Table 2:** Distribution of positive carbapenemase gene loci according to bacteria types

Genes	Type of bacteria n (%)											Total
	*K. pneumoniae*	*S. marcescens*	*M. morganii*	*P. mirabilis*	*E. coli*	*E. cloacae*	*E. aerogenes*	*C. freundii*	*R. planticola*	*P. stuartii*	*K. oxytoca*	
OXA-48	52 (75.36)	5 (15.15)	3 (17.65)	6 (35.29)	8 (61.54)	3 (23.08)	5 (50)	1 (50)	1 (50)	1 (100)	1 (100)	86 (47.51)
NDM-1	5 (7.25)	0	0	0	0	1 (7.69)	0	0	0	0	0	6 (3.31)
VIM	1 (1.45)	0	0	0	0	0	0	0	0	0	0	1 (0.55)

MEM-MHT was positive in 78 of the 86 (90.69 %) bla_OXA-48_ positive isolates. ERT-MHT was positive in 77 of the 86 (89.53 %) bla_OXA-48_ positive isolates. All NDM and VIM positive isolates were positive with MHT and E-test MBL. NDM-1-positive *E. cloacae* were both MHT and E-test MBL positive. MEM-MHT, ERT-MHT results according to different OXA-48 positive CRE isolates was shown in Table 3. Sensitivity, specificity, negative predictive value (NPV) and positive predictive value (PPV) of MEM-MHT and ERT-MHT for bla_OXA-48_ positive isolates were given in Table 4.

**Table 3:** Distribution of MEM-MHT, ERT-MHT results according to carbapenem- resistant *Enterobacteriaceae* isolates

	Total number	Number of MEM-MHT (+) isolates	Number of MEM-MHT (−) isolates	Number of ERT-MHT(+) isolates	Number of ERT-MHT(−) isolates
All isolates	181	110	71	109	72
OXA-48 positive isolates	86	78	8	77	9
*K. pneumoniae*	69	56	13	55	14
OXA-48 positive *K. pneumoniae*	52	50	2	51	1
*S. marcescens*	33	10	23	6	27
OXA-48 positive *S. marcescens*	5	2	3	1	4
*M. morganii*	17	8	9	9	8
OXA-48 positive *M. morganii*	3	3	0	3	0
*P. mirabilis*	17	9	8	10	7
OXA-48 positive *P. mirabilis*	6	4	2	4	2
*E. coli*	13	8	5	10	3
OXA-48 positive *E. coli*	8	8	0	8	0
*E. cloacae*	13	8	5	8	5
OXA-48 positive *E. cloacae*	3	3	0	3	0
*E. aerogenes*	10	7	3	7	3
OXA-48 positive *E. aerogenes*	5	5	0	5	0
*C. freundii*	2	0	2	1	1
OXA-48 positive *C. freundii*	1	0	1	0	1
*R. planticola*	2	1	1	0	2
OXA-48 positive *R. planticola*	1	1	0	0	1
*P. stuartii*	1	1	0	1	0
OXA-48 positive *P. stuartii*	1	1	0	1	0
*K. oxytoca*	1	1	0	1	0
OXA-48 positive *K. oxytoca*	1	1	0	1	0

*MEM-MHT* modified Hodge test with meropenem, *ERT-MHT* modified Hodge test with ertapenem

**Table 4:** Sensitivity, specificity, negative predictive value, positive predictive value of MEM-MHT, ERT-MHT and correlation with OXA-48 gene positivity

		Sensitivity %	Specificity %	NPV %	PPV %	Correlation with OXA-48 gene positivity
All OXA-48 positive isolates	MEM-MHT	70.9	88.7	66.3	90.7	p < 0.001
	ERT-MHT	70.6	87.5	66.3	89.5	p < 0.001
OXA-48 positive *K. pneumoniae*	MEM-MHT	89.3	84.6	64.7	96.2	p < 0.001
	ERT-MHT	92.7	92.9	76.5	98.1	p < 0.001
OXA-48 positive *S. marcescens*	MEM-MHT	20	86.9	71.4	40	p > 0.05
	ERT-MHT	16.7	85.2	82.1	20	p > 0.05
OXA-48 positive *M. morganii*	MEM-MHT	37.5	100	64.3	100	p > 0.05
	ERT-MHT	33.3	100	57.1	100	p > 0.05
OXA-48 positive *P. mirabilis*	MEM-MHT	44.4	75	54.6	66.7	p > 0.05
	ERT-MHT	40	71.4	45.5	66.7	p > 0.05
OXA-48 positive *E. coli*	MEM-MHT	100	100	100	100	p < 0.001
	ERT-MHT	80	100	60	100	p < 0.05
OXA-48 positive *E. cloacae*	MEM-MHT	37.5	100	50	100	p > 0.05
	ERT-MHT	37.5	100	50	100	p > 0.05
OXA-48 positive *E. aerogenes*	MEM-MHT	71.43	100	60	100	p > 0.05
	ERT-MHT	71.43	100	60	100	p > 0.05

*MEM-MHT* modified Hodge test with meropenem, *ERT-MHT* modified Hodge test with ertapenem, *NPV* negative predictive value, *PPV* positive predictive value

There was a statistically significant correlation between bla_OXA-48_ gene positivity and MEM-MHT positivity (p < 0.001). There was also a statistically significant correlation between bla_OXA-48_ gene positivity and ERT-MHT positivity (p < 0.001). MEM-MHT was found highly discriminative for bla_OXA-48_-positive *E. coli* (p < 0.001). Accuracy rate of MEM-MHT and ERT-MHT were not good for other CRE species. High rate of false positive and negative results were observed.

The sensitivity of E-test MBL for bla_NDM-1_ positive isolates was 66.7 %, specificity was 100 %, NPV 98.3 %, PPV 100 %. There was a statistically significant correlation between bla_NDM-1_ positivity and E-test MBL positivity (p < 0.001). When bla_NDM-1_ positive and bla_VIM_ positive isolates were considered together sensitivity of E-test MBL was 77.8 %, specificity was 100 %, NPV 98.9 %, PPV 100 %.

Carbapenemase genes were detected in 31 (36.05 %) strains isolated from urine samples, 21 (24.42 %) strains isolated from blood samples, 16 (18.60 %) strains isolated from tracheal aspirate samples, 11 (12.79 %) strains isolated from wound samples, 4 (4.65 %) strains from abscess samples, 3 (3.49 %) strains isolated from catheter samples, 1 (1.16 %) strain from sputum, 1 (1.16 %) strain from peritoneal fluid, 1 (1.16 %) strain from isolated from bile.

Carbapenemase genes were detected mostly in 18 (20.93 %) samples isolated from patients from internal ICU. This was followed by 13 (15.12 %) samples isolated from PACU patients, 9 (10.47 %) samples from surgical ICU, 6 (6.77 %) samples from urology patients, 5 (5.81 %) samples from emergency internal medicine patients, 4 (4.65 %) samples from neurosurgery patients 4 (4.65 %) samples from general surgery patients, 4 (4.65 %) samples from emergency medicine patients and 23 samples from other hospital units.

### Isolation of multiple CRE from the same patient

In two patients more than one genus of CREs was isolated, and the bla_OXA-48_ gene was positive in all these isolates. First was a patient from surgical ICU. OXA-48-positive *K. pneumoniae* was isolated from wound sample then 2 days later OXA-48-positive *K. oxytoca* was isolated from tracheal aspirate and then 4 days later OXA-48-positive *E. coli* was isolated from an abscess. The second was a patient from bone marrow transplantation (BMT) unit. An OXA-48-positive *E. cloacae* was isolated from urine and 9 days later OXA-48-positive *E. coli* was isolated from blood.

### Antimicrobial susceptibility results of carbapenemase negative and positive CRE

When all 181 CRE isolates were considered; 95.58 % were resistant to AMP, 91.16 % were resistant to AMC, 80.11 % were resistant to TZP, 80.11 % were resistant to CXM, 82.32 % were resistant to CAE, 76.80 % were resistant to FOX, 60.22 % were resistant to CAZ, 68.51 % were resistant to CRO, 51.38 % were resistant to FEP, 55.80 % were resistant to ERT, 94.47 % were resistant to IPM, 44.75 % were resistant to MEM, 23.20 % were resistant to AMK, 39.23 % were resistant to GEN, 46.96 % were resistant to CIP, 46.41 % were resistant to CST, 38.67 % were resistant to SXT. Antimicrobial resistance results of each species were shown in Table 5.

**Table 5:** Antibiotic resistance rates of most commonly isolated carbapenem-resistant *Enterobacteriaceae* species in our study

Species	Antibiotic resistance rate (%)																
	AMP	AMC	TZP	CXM	CAE	FOX	CAZ	CRO	FEP	ERT	IPM	MEM	AMK	GEN	CIP	CST	SXT
*K. pneumoniae*	100	100	100	88.41	88.41	85.51	86.96	86.96	86.96	100	92.75	78.26	46.38	63.77	78.26	14.49	55.07
*S. marcescens*	100	100	30.30	100	100	100	30.30	100	6.06	0	100	0	3.03	0	0	100	0
*M. morganii*	100	100	52.94	100	100	52.94	58.82	41.18	58.82	0	100	11.76	17.65	11.76	29.41	100	47.06
*P. mirabilis*	52.94	29.41	17.65	0	0	0	23.53	0	17.65	0	100	5.88	0	29.41	35.29	100	41.18
*E. coli*	100	92.31	92.31	92.31	84.62	76.92	69.23	76.92	76.92	84.62	84.62	76.92	15.38	53.85	61.54	7.69	61.54
*E. cloacae*	100	100	69.23	76.92	100	100	61.54	61.54	38.46	84.62	92.31	61.54	30.77	61.54	61.54	7.96	53.85
*E. aerogenes*	100	100	50	50	60	100	40	30	0	50	90	30	0	0	10	10	0

*AMC* amoxicillin-clavulanic acid, *AMK* amikacin, *AMP* ampicillin, *CAE* cefuroxime-axetil, *CAZ* ceftazidime, *CIP* ciprofloxacin, *CRO* ceftriaxone, *CST* colistin, *CXM* cefuroxime, *ERT* ertapenem, *FEP* cefepime, *FOX* cefoxitin, *GEN* gentamicin, *IPM* imipenem, *MEM* meropenem, *SXT* trimethoprim-sulfamethoxazole, *TZP* piperacillin-tazobactam

Ninety-two of the 181 isolates (50.8 %) were multidrug-resistant which was defined as being resistant to three or more functional classes of drugs simultaneously by Magiorakos et al. [21]. Fifty-one of the 69 *K. pneumoniae* isolates were found to be multidrug-resistant, and four of them were found to be resistant to all tested antibiotics. Eight of the 13 *E. coli,* 10 of 17 *P. mirabilis*, eight of 17 *M. morganii*, eight of the 13 *E. cloacae*, one the 10 *E. aerogenes,* two of the two *C. freundii*, one the two *R. planticola,* one of one *P. rettgeri*, one of one *P. stuartii*, and only one of the 33 *S. marcescens* were multidrug-resistant. Fifty three (61.62 %) of 86 OXA-48 positive isolates and 39 (41.05 %) of the 95 OXA-48 negative isolates were multidrug-resistant. There were more multidrug-resistant isolates in OXA-48 positive isolates than OXA-48 negative ones, and this difference was statistically significant (p < 0.01). All metallo-β-lactamase (NDM-1, VIM) positive isolates were found to be multidrug-resistant (p < 0.01). There was a statistically significant correlation between having one of the carbapenemase genes and being multidrug-resistant (p < 0.001).

Most of the *K. pneumoniae, E. coli* and *E. cloacae* isolates were resistant to carbapenems, beta-lactam/beta-lactamase inhibitor combinations, third and fourth generation cephalosporins, CIP and SXT. Thirty-seven out of 69 *K.pneumoniae* were found to be susceptible to AMK, and 25 were susceptible to GEN. Ten of the *K. pneumoniae* were found to be resistant to CST. One *E.cloacae* and one *E.coli* isolate were found to be resistant to CST. Twenty-three of *K. pneumoniae* isolates were identified as ESBL producers.

Fifty-four of the 69 *K. pneumoniae* (78.26 %) had a least a carbapenemase gene. Eighteen of the carbapenemase-positive *K. pneumoniae* were also ESBL positive. Both carbapenemase-negative and carbapenemase-positive *K. pneumoniae* were found to be 100 % resistant to AMP, AMC, TZP and ERT. Carbapenemase-positive *K. pneumoniae* was found to be slightly more resistant to FOX (87.04 vs 80 %), CAZ (87.04 vs 86.67 %), CRO (87.04 vs 86.67 %), FEP (87.04 vs 86.67 %), IPM (96.30 vs 86.67 %), MEM (79.63 vs 73.33 %), GEN (64.81 vs 60 %), CST (16.67 vs 6.67 %) than carbapenemase-negative *K. pneumoniae*. On the other hand carbapenemase-negative *K. pneumoniae* were more resistant to CXM (93.33 vs 88.89 %), CAE (93.33 vs 88.89 %), AMK (66.67 vs 40.74 %), CIP (80 vs 77.78 %), and SXT (73.33 vs 50 %) than carbapenemase-positive *K. pneumoniae.* However, these differences were not statistically significant (p > 0.05).

One of the OXA-48 and NDM-1 positive *K.pneumoniae* was found resistant to all of the tested antibiotics.

Three *E.coli* isolates were ESBL positive. Eight of the OXA-48 positive isolates were found to be multidrug-resistant. Both OXA-48 positive and negative isolates were found to be 100 % resistant to AMP. *E. coli* which posses bla_OXA-48_ gene were found to be more resistant to antibiotics than bla_OXA-48_ negative *E. coli*: AMC (100 vs 80 %), TZP (100 vs 80 %), CXM (100 vs 80 %), CAE (100 vs 60 %), FOX (100 vs 40 %), CAZ (100 vs 20 %), CRO (100 vs 40 %), FEP (100 vs 40 %), ERT (100 vs 60 %), IPM (100 vs 60 %), MEM(100 vs 40 %), AMK (25 vs 0 %), GEN (62.5 vs 20 %), CIP (75 vs 40 %), SXT (62.5 vs 60 %) except for CST (0 vs 20 %). Only the differences for FOX, CAZ, CRO, FEP, MEM were statistically significant (p < 0.05).

One isolate of *E. cloacae* was found to produce bla_NDM-1_. This isolate was found to be highly resistant to tested antibiotics with high MICs and found to be susceptible to only to CST. It was isolated from the urine sample of a patient who was residing at internal medicine unit. When patient’s history was examined, there was no history of foreign travel. This patient had a previous history of use of cephalosporins and carbapenems. Similarly, none of the other patients with bla_NDM-1_ gene positive CRE infections had records of foreign travel.

*S. marcescens* isolates were mostly resistant to beta-lactam/beta-lactamase inhibitor combinations, second and third generation cephalosporins. All were resistant to IMP, and all were resistant to CST. All were susceptible to ERT, MEM, GEN, CIP and SXT.

*M. morganii* isolates were mostly resistant to beta-lactam/beta-lactamase inhibitor combinations, IMP and CST. Nearly half of the *M. morganii* isolates were resistant to FOX, CAZ, CRO, FEP and SXT. All *M. morganii* isolates were susceptible to ERT. Two of the isolates were resistant to MEM. Three of the isolates were resistant to AMK, two were resistant to GEN, and five were resistant to CIP.

*P. mirabilis* isolates were mostly susceptible to beta-lactam/beta-lactamase inhibitor combinations, cephalosporins and aminoglycosides. All *P. mirabilis* isolates were found to be susceptible to ERT, and only one was resistant to MEM. All *P. mirabilis* isolates were resistant to IMP and CST.

All *E. aerogenes* isolates were susceptible to FEP, aminoglycosides (AMK and GEN) and SXT. Only one *E. aerogenes* was found to be resistant to CST, and one was resistant to CIP.

In our study *K. pneumoniae, E. coli, E. cloacae* isolates were found highly resistant to ERT, IPM and MEM. *S. marcescens*, *M. morganii*, *P. mirabilis* were mostly susceptible to ERT but highly resistant to IPM. CST was found to be most effective antibiotic against *K. pneumoniae, E. coli, E. cloacae*. On the other hand, *S. marcescens, M. morganii, P. mirabilis, P. vulgaris, P. stuartii, P. rettgeri, C. sakazakii* were found to be 100 % resistant to CST. When 181 CRE are considered AMK (23.20 % resistant) and SXT (38.67 % resistant) were found as most effective antibiotics. Resistance to CST was 46.41 % overall but when the resistance of species with intrinsic resistance were excluded (*Serratia, Morganella, Proteus*, etc.) it was 12.61 %.

## Discussion

CRE are increasingly isolated from community-acquired and nosocomial infections [6]. CRE can spread clonally from person to person or genes which encode carbapenemases can spread horizontally between isolates [2]. This is an important health concern because plasmid-encoded and easily transferable carbapenemases are involved [10]. The most important carbapenemases are KPC, VIM, NDM and OXA-48 [6]. CRE infections are highly mortal, and treatment options are narrow [7, 10]. Rapid identification of carbapenemase-producing strains is crucial for preventing hospital infections and outbreaks [7].

Certain risk factors were found to be related to the acquirement of CRE infections. These situations are being admitted to ICU, central venous catheter presence, usage of antibiotic such as cephalosporins, having diabetes mellitus, having surgical procedures, long stay in the hospital and being a transplant patient [7]. In our study majority of our CRE strains were isolated from ICUs.

The rate of CRE in our hospital during the study period was found as 2.82 %. In the United States prevalence of CRE was found to be between 1.4–4.2 % [2]. Similar CRE isolation rates to our study have been reported from many Asian countries: 1.2 % in Lebanon, 4.05 % from Malaysia, <1 in Singapore, 1.2 % in Taiwan, 1.17 % in Saudi Arabia [4, 5].

The most common CRE species isolated in the US and European countries are *K. pneumoniae* followed by *E. aerogenes* and *E. coli* [2, 18]. In accordance to this in our study, the most common isolated species was *K. pneumoniae.* But our second most was *S. marcescens* and third most were *M. morganii* and *P. mirabilis.*

Our country has a geographical importance for isolation of carbapenemase-producing bacteria. However molecular studies regarding carbapenemase resistance are limited especially for CRE other than CRKP [7].

After its initial identification from a *K. pneumoniae* strain in 2001 in Turkey, OXA-48 producers have become increasingly important causes of nosocomial outbreaks in our country [22, 23]. Today OXA-48 producing *Enterobacteriaceae* have spread to Middle East (Lebanon, Israel), and North African countries (Morocco, Tunisia, Egypt, Libya, Algeria), Mediterranean countries (Greece, Spain, Italy, France, Slovenia, Croatia), and European countries (Germany, Belgium, Switzerland, the Netherlands, the UK), North America (USA), South America (Argentina) and Asia (India, Taiwan) [7, 10, 17, 20, 22, 23]. The bla_OXA-48_ gene in *Enterobacteriaceae* is located between two identical copies of IS*1999*, which altogether forms composite transposon Tn*1999* on a mobilizable, self-conjugative plasmid [4, 7, 8, 10, 14, 17, 22–25] In previous studies it was shown that bla_OXA-48_-carrying plasmids can transfer both clonally and horizontally [25].

Initially and most commonly bla_OXA-48_ gene was identified in *K. pneumoniae*. But currently, many other species of *Enterobacteriaceae* are known to possess them, such as *E. coli*, *Enterobacter* spp., *K. oxytoca*, *C.freundii*, *S. marcescens*, *M. morganii*, *P. mirabilis* [17, 25]. In our study majority of OXA-48 producing isolates were *K. pneumoniae*. There is an ongoing epidemic of bla_OXA-48_ positive *K. pneumoniae* nosocomial infections in our hospital since 2010 (personal data). We guess spread to other *Enterobacteriaceae* species was occurred from these *K. pneumoniae* isolates. Interestingly, during our study period more than one genus of bla_OXA-48_ gene positive CRE were isolated in two patients. This suggests horizontal interspecies dissemination. The bla_OXA-48_-like genes were known to be located on conjugative plasmids of IncL/M incompatibility group which can transfer between different species of *Enterobacteriaceae* [25]. The bla_OXA-48_ can successfully spread to different enterobacterial species with the help of high transfer efficiency of the plasmids that carry this gene [23]. This explains the interspecies transmission of the bla_OXA-48_ gene.

Arana et al. have reported they have recovered four different CRE species (*K. pneumoniae, E. coli, S. marcescens, C. freundii*) from a patient hospitalized for 4 months. These bacteria all had the bla_OXA-48_ gene. They mentioned the great ability of OXA-48 carbapenemase to spread among different enterobacterial species [26].

In our country, the bla_OXA-48_ genes were identified previously in *K. pneumoniae*, *E. aerogenes, E. coli*, *C. freundii*, *E. cloacae*, *S.marcescens*, *P. rettgeri, K. oxytoca* [24, 27, 28]. To our knowledge, this is the first report of bla_OXA-48_ genes identified in *P. mirabilis*, *M. morganii*, *P. stuartii*, *R. planticola* in Turkey.

The OXA-48 enzyme can successfully hydrolyze penicillins but have poor or no activity against extended-spectrum cephalosporins and aztreonam. They also have a weak carbapenemase activity and cannot show a high level of carbapenem resistance. The contribution of other factors such as the production of ESBLs or cell wall permeability defects are necessary for the increased level of resistance to cephalosporins and carbapenems [10, 20, 22–26]. In other studies, a close association was found between OXA-48 enzyme production and expression of ESBLs (CTX-M-15, SHV) [8, 10, 13, 17, 20, 24]. Co-expression of OXA-48 and AmpC enzymes in CRE were demonstrated, previously [1].

In our study majority of OXA-48 positive isolates were resistant to extended-spectrum cephalosporins and had high levels of resistance to carbapenems. These findings suggest that other mechanisms of resistance are present in our isolates.

On the other hand, metallo-β-lactamases (IMP, VIM, and NDM) can yield high levels of resistance to carbapenems. But they cannot hydrolyze aztreonam. They can be inhibited by EDTA [1, 10, 12]. E-test MBL strips use the principle of inhibition of metallo-β-lactamases by EDTA when IPM is used as a substrate. Doyle et al. said that it is often difficult to interpret E-test MBL for investigation of metallo-β-lactamases in *Enterobacteriaceae* because MIC values of IPM are low in metallo-β-lactamases -producing *Enterobacteriaceae*. They found that sensitivity of metallo-β-lactamases was low, but specificity was high [15]. However, in our study, we found nine isolates positive with E-test MBL and seven of these isolates had a metallo-β-lactamases gene. The sensitivity and specificity of E-test MBL for metallo-β-lactamases in our study was 77.8 and 100 %, respectively. The false positive results may be due to increased outer membrane permeability caused by EDTA used in the test [29].

Verona integron encoded metallo-β-lactamase (VIM) was first reported from Italy in 1997 [23]. *Enterobacteriaceae* producing VIM enzymes are found prevalently in Mediterranean countries. VIM -1- producing *Enterobacteriaceae* are endemic in Greece [10, 22, 23]. In Turkey the first detection of VIM-1 was from a *K. pneumoniae* isolate from the urine of three-year-old girl in 2005 by Yıldırım et al. [30]. In our study, we identified bla_VIM_ in a *K. pneumoniae* isolate which also had the bla_OXA-48_ gene. This strain was resistant to all tested antibiotics (including CST) except SXT. VIM enzymes are found to be associated with class1 integrons that carry gene cassettes which encode resistance genes to various antibiotics [22].

Another plasmid-mediated metallo-β-lactamases, New Delhi metallo-β-lactamase-1 (NDM-1) was first discovered in a *K. pneumoniae* strain isolated from the urine sample of a Swedish patient who was previously hospitalized in India in 2009 [3, 10, 22, 23]. Later it spread to Balkans and Middle East [1]. NDM-producing *Enterobacteriaceae* are widespread in India, Pakistan, and Bangladesh [22]. The first report of NDM-1 from Turkey was in 2011 by Poirel et al. from a *K. pneumoniae* strain isolated from an allogenic stem cell-transplanted leukemia patient who was previously hospitalized in Iraq [7]. The second case report in Turkey was from our laboratory in 2013 from a *K. pneumoniae* isolated from the blood culture of a Syrian patient [31]. In the present study, we detected bla_NDM-1_ gene in six of the CRE isolates (five *K.pneumoniae* and one *E. cloacae*). Three of the bla_NDM-1_ positive *K.pneumoniae* isolates were also positive for the bla_OXA-48_ gene. We have found that all six bla_NDM-1_ positive isolates were resistant to all β-lactam antibiotics, AMK, GEN, CIP, and SXT. Two of the bla_NDM-1_ positive *K. pneumoniae* isolates were found to be resistant to CST.

In previous studies, similar to our findings NDM-1 producing *Enterobacteriaceae* are found to be often broadly resistant to many other drug classes besides β-lactams. NDM-1 producing isolates may carry a diversity of other resistance mechanisms such as β-lactamases (AmpC cephalosporinases, CTX-M, CMY), other types of carbapenemases (OXA-48, KPC and VIM), enzymes that cause resistance to aminoglycosides (16S RNA methylase), to quinolones (Qnr), to macrolids (esterases), to rifampicin (rifampicin-modifying enzymes), to chloramphenicol and SXT, to CST [2, 10, 22, 23].

NDM-1 was most commonly identified in *K. pneumoniae* and *E. coli* isolates but has been increasingly reported in other species of *Enterobacteriaceae* including *C. freundii*, *M. morganii*, *E. cloacae*, *K.oxytoca* [2, 3, 6, 22]. This may be because NDM enzymes are encoded on highly mobile, conjugative plasmids which facilitate horizontal inter- and intraspecies transfer between bacteria rather than clonal spread [2, 3, 10]. We have found one NDM-1 producing *E. cloacae* in our study. Previously Poirel et al. reported an NDM-1-producing *E. cloacae* outbreak in a neonatal ICU in Turkey [32]. NDM-1 producing *E. cloacae* previously have been defined as one of the most commonly reported CRE species and known to cause hospital outbreaks in many other parts of the world [3]. In another study from our country, Demir et al. isolated NDM-1 positive *S. marcescens* and *K. oxytoca* [6].This is particularly concerning because NDM isolates are highly resistant to many classes of antibiotics and have the potential to spread rapidly to many members of *Enterobacteriaceae* and cause nosocomial outbreaks.

The first identified cases of NDM-producing CRE in our country had histories of foreign country travel [7, 31]. None of the NDM cases in our study had a history of foreign travel. Similar to our findings in two studies from our country conducted by Alp *et al.* and Sahin *et al.* no international contact history was found in patients with NDM-1-producing CRE [7, 33]. This is important because it means NDM-1 carbapenem resistance is spreading in Turkey.

The development of methods for rapid and accurate identification of CRE is necessary for appropriate selection of antibiotic treatment and prevention of hospital outbreaks [7, 9]. Automated antibiotic susceptibility systems were found to be unreliable for detection of carbapenem resistance. These automated systems either over or under reported carbapenem resistance [2, 12]. More practical molecular methods are needed. In this study, we used a hybridization-based multiplex PCR method which was reported to have 99 % specificity for OXA-48 and 100 % for other enzymes [34]. Two other studies from our country reported to use this multiplex PCR method and get accurate results [6, 7]. Demir et al. analyzed 95 *Enterobacteriaceae* spp. isolates with this system and found bla_OXA-48_ in 49 isolates, bla_NDM-1_ in six isolates and bla_VIM_ in two isolates. They did not find any carbapenemase genes in carbapenemase susceptible strains [6]. Sahin et al. found seven bla_OXA-48_ positive strain and one bla_NDM-1_ positive strain when they studied 43 CRE isolate [7].

MHT is known be reliable for detection of KPCs and OXA-48-like enzymes but perform poorly for metallo-β-lactamases (NDMs, VIMs, and IMPs) [2, 9, 12, 15, 24]. In a study Doyle et al. found MHT had the sensitivity of 98 % for detection of KPC-producers, 93 % for OXA-48-like producers and only 12 % for metallo-β-lactamase producers [15]. However, we found that all metallo-β-lactamase (NDM-1 and VIM) positive bacteria in our study were MHT positive.

Another shortcoming of MHT is it can give false-positive results for carbapenem-resistant but noncarbapenemase-producing strains [15]. Also, there is limited data on MHT’s performance for CRE other than carbapenem-resistant *K. pneumoniae* and *E. coli*. Hammoud et al. showed MHT gave false-positive results in *E. cloacae* [4]. In this regard, our study was the first study that analyzed performance of MHT on many different CRE species. The sensitivity of MEM-MHT for bla_OXA-48_ positive isolates was found 70.9 %; specificity was 88.7 %, sensitivity of ERT-MHT for bla_OXA-48_ positive isolates was 70.6 %, specificity was 87.5 %. A good correlation was found between bla_OXA-48_ and MHT positivity.

In our study MHT performed gave best results for OXA-48-producing *E. coli.* MHT’s results for *K. pneumoniae* were acceptable. However, MHT performed poorly for *S. marcescens*, *M. morganii, P. mirabilis, E. cloacae, E. aerogenes.*

MHT gave false positive and negative results for OXA-48-producing *K. pneumoniae*, *S. marcescens*, *P. mirabilis*, *and C. freundii*. MHT gave false positive results for OXA-48-producing *M. morganii*, *E.cloacae*, *E. aerogenes*.

In many studies, tigecycline and CST were found as most effective antimicrobial agents against CRE [1, 5, 24, 25]. In our study when antibiotic resistance profiles of all isolates were considered together, AMK (23.20 %) was found as most effective antibiotic, followed by SXT (38.67 %). Similar to our findings Wang et al. found that AMK susceptibility was very high among the CRE in their study [1]. Pollet et al. found that AMK and GEN were the most effective antibiotics followed by SXT when cumulative antibiogram of 115 CRE isolates was studied. In that study, CST and tigecycline were not tested [2]. CST resistance rate was high in our study because of the strains with intrinsic CST resistance were included in cumulative antibiotic resistance profile. On the other hand, CST was found as the most effective antibiotic against non-intrinsically CST resistant CRE strains (*K. pneumoniae*, *E. coli*, *E.cloacae*, *E. aerogenes*, *R. planticola*, *K. oxytoca*).

We have found that 92 of the 181 isolates were multidrug-resistant. The number of multidrug-resistant strains in OXA-48 producing isolates were slightly more than OXA-48 negative ones, and this difference was statistically significant. All metallo-β-lactamase positive isolates were multidrug-resistant. In previous studies, it was shown that the same plasmids that carry carbapenemase genes can often carry resistance genes to other antibiotics, and the rate of multidrug resistance among CRE is high [14, 24, 25].

Hammoudi et al. in their study found that OXA-48 positive *S. marcescens* remained susceptible to most tested aminoglycosides and fluoroquinolones while all the remaining CRE that they studied were multidrug-resistant [14]. Similar to this *S. marcescens* isolates in our study were found susceptible to AMK, GEN, CIP, and SXT. Only one *S. marcescens* in our study was multidrug-resistant. Similar to this *E. aerogenes* strains in our study were mostly susceptible to AMK, GEN, CIP, SXT and CST and only one of *E. aerogenes* strains was multidrug-resistant.

Fursova et al. showed that most of the CRE strains they studied were multidrug-resistant (98.9 % of *K. pneumoniae* and 100 % of *P. mirabilis*). They found that most active antibiotic against *K. pneumoniae* was CST and most effective antibiotics against *P. mirabilis* were cefoperazone/sulbactam, ERT, and CAZ [25]. Similar to this we found most effective antibiotics against carbapenem-resistant *P. mirabilis* were CXM, CAE, FOX, CRO, ERT, and AMK.

## Conclusions

The findings of our study indicate that OXA-48 resistance is spreading rapidly throughout many *Enterobacteriaceae* species in Turkey. Also, NDM-1 isolates without a history of foreign contact are increasingly being found in our country. This is alarming. Therefore, carbapenem resistance mechanisms in *Enterobacteriaceae* should be rapidly determined and infection control precautions should be immediately taken.

## Abbreviations

AMC: amoxicillin-clavulanic acid
AMK: amikacin
AMP: ampicillin
AmpC: AmpC beta-lactamases
ATCC: American Type Culture Collection
BMT: bone marrow transplantation
CAE: cefuroxime-axetil
CAZ: ceftazidime
CIP: ciprofloxacin
CLSI: Clinical and Laboratory Standards Institute
CPE: carbapenemase-producing *Enterobacteriaceae*
CRE: carbapenem-resistant *Enterobacteriaceae*
CRKP: carbapenem-resistant *Klebsiella pneumonia*
CRO: ceftriaxone
CSF: cerebrospinal fluid
CST: colistin
CXM: cefuroxime
DNA: deoxyribonucleic acid
EDTA: ethylene diamine tetra-acetic acid
ELISA: enzyme linked immunosorbent assay
ERT: ertapenem
ERT-MHT: modified Hodge test with ertapenem
ESBL: extended-spectrum β-lactamases
E-test: epsilometer test
FEP: cefepime
FOX: cefoxitin
GEN: gentamicin
GES: Guiana extended spectrum
ICU: intensive care unit
IMP: IMP type metallo-beta-lactamase
IPM: imipenem
KPC: Klebsiella pneumoniae carbapenemase
MALDI: matrix assisted laser desorption ionisation
MBL: metallo-β-lactamase
MEM: meropenem
MEM-MHT: modified Hodge test with meropenem
MHT: modified Hodge test
MIC: minimal inhibitory concentration
NDM-1: New Delhi metallo-β-lactamase-1
NmcA: not metalloenzyme carbapenemase
NPV: negative predictive value
OXA: oxacillinase
PACU: post anesthesia care unit
PCR: polymerase chain reaction
PPV: positive predictive value
RNA: ribonucleic acid
SXT: trimethoprim-sulfamethoxazole
TZP: piperacillin-tazobactam
VIM: Verona integron encoded metallo-β-lactamase
